# Cooking, storage, and reheating effect on the formation of cholesterol oxidation products in processed meat products

**DOI:** 10.1186/s12944-015-0091-5

**Published:** 2015-08-11

**Authors:** Muhammad I. Khan, Joong-Seok Min, Sang-Ok Lee, Dong Gyun Yim, Kuk-Hwan Seol, Mooha Lee, Cheorun Jo

**Affiliations:** Department of Agricultural Biotechnology, Center for Food and Bioconvergence, and Research Institute of Agriculture and Life Science, Seoul National University, Seoul, 151-921 South Korea; National Institute of Food Science and Technology, University of Agriculture, Faisalabad, 38040 Pakistan; CJ Food Research Center, Seoul, 152-050 Korea; Department of Health Administration and Food Hygiene, Jinju Health College, Jinju, 660-757 South Korea; National Institute of Animal Science, RDA, Cheonan, 331-801 South Korea; College of Agriculture and Life Science, Arsi University, Asella, Ethiopia

**Keywords:** Processed meat products, Cooking and reheating methods, Total cholesterol, Cholesterol oxidation products (COPs)

## Abstract

**Background:**

Cholesterol is an important biological compound; however, its oxidation products have been proven to be harmful to human health. Cooking, storage, and reheating methods significantly affect the safety of meat products, as they contribute to the production of cholesterol oxidation products (COPs).

**Methods:**

Three cooking methods were used to cook sausages, loin ham, bacon, luncheon meat, and pressed ham, in order to investigate the effect of cooking, storage, and reheating on total cholesterol and on the formation of COPs. Cooked samples were stored at 4 °C and reheated after 3 and 6 storage days by the same cooking method or by microwaving. The samples were assessed for total lipids, cholesterol, and cholesterol oxides.

**Results:**

The average cholesterol content in the processed meat varied from 76.0 mg/100 g to 201.70 mg/100 g. Microwaved ham showed the lowest cholesterol content compared to that of other processed meat products. Significant differences were found in cholesterol content and cholesterol oxidation products depending on cooking, storage, and reheating methods. Six cholesterol oxides were found in processed meat, of which 7β-hydroxycholesterol and α-epoxides were detected as the major oxidation products.

**Conclusions:**

Microwaving and oven grilling resulted in higher production of COPs in processed meat as compared with other cooking methods. Refrigerated storage tended to significantly increase the COPs content.

## Background

During the last two decades, the relationship between diet and health has been widely studied and consumers were encouraged to improve their dietary habits. However, the intake of fats, namely saturated fats, is still higher than that specified by the American Heart Association recommendations, according to which total fats should not exceed 30 % of the total caloric intake and saturated fats should represent less than 10 % of the total calories. Processed meat products are highly appreciated by a large number of populations; however, they are rich in cholesterol, lipids, and saturated fatty acids. In general, fats of animal origin are not considered healthy because of their high content of saturated fatty acids and cholesterol [1]. Thermal processing of meat and meat products leads to undesirable changes such as lipid oxidation and protein degradation [2]. Excessive oxidation of meat lipids produces potential precursors of highly reactive aldehydes in tissues and foods, becoming a source of oxidative stress [3, 4]. These aldehydes can be major contributing factors in several pathological conditions such as atherosclerosis, inflammation, arthritis, Alzheimer’s, and Parkinson’s disease [5]. Healthy human plasma contains 12.6 mg/L of COPs [6] and ingestion of foods containing COPs increases these levels in plasma and leads to deleterious health effects.

Cholesterol is a compound of biological importance, widely distributed in food of animal origin. Cholesterol contents of meat and meat products varied considerably but general it is less than 70 mg/100 g except for edible offal and it is assumed that one-third of daily intake come from meat and meat products [7]. However, cholesterol oxidation products (COPs) have been proven to be cytotoxic, mutagenic, and carcinogenic [8], and are also considered to be a primary factor responsible for triggering atherosclerosis [9]. COPs are formed when animal-derived foods are subjected to heating and cooking [10], dehydration [11] storage [1], and irradiation [12]. Colorimetric, chromatographic and enzymatic assessment methods are used for assessing cholesterol and its products [13]. Dominguez et al. [14] reported that, among different cooking methods, i.e., roasting, grilling, microwaving, and frying, used for cooking foal meat, microwaved samples showed the highest oxidation products. Lee et al. [12] studied the effects of various cooking and reheating methods on the total cholesterol and on the formation of COPs in beef loin, and observed a significant reduction in cholesterol and an increase in COPs. Hu et al. [15] compared the effects of cooking methods on pork lipid digestibility and on the formation of COPs, and detected a significantly higher COP formation in microwave-treated samples. In a recent study, Freitas et al. [16] reported the reduction in cholesterol content and the increase in COPs, especially 7-ketocholesterol, in fish fillets cooked by different methods.

Hence, this study aimed to determine the effects of cooking methods, storage, and reheating on the amount of total lipids and cholesterol, and on the formation of COPs in processed meat products.

## Methods

### Reagents and solutions

Cholesterol, linoleic acid, oleic acid, cholesterol oxide standards [7-ketocholesterol (7-keto), 6-ketocholesterol (6-keto), 7α-hydroxycholesterol (7α-OH), 7β-hydroxycholesterol (7β-OH), 5,6α-epoxycholesterol (5,6α-EP), 5,6β-epoxycholesterol (5,6β-EP), 25-hydroxycholesterol (25-OH), 20-hydroxycholesterol (20-OH), and cholestanetriol (triol)], butylated hydroxytoluene (BHT), Xpyridine, and silicic acid (100 mesh) were purchased from Sigma-Aldrich Co., LLC (Seoul, Korea). Bis(trimethylsilyl)trifluoroacetamide (BSTFA) + 1 % trimethylchlorosilane (TMCS) was obtained from Supelco (Bellefonte, PA, USA). HPLC-grade hexane, ethyl acetate, acetone, methanol, chloroform, Celite 545, and calcium phosphate (CaHPO_4_^.^2H_2_O) were purchased from Fisher Scientific Co. (Malvern, PA, USA).

### Sample preparation

Five processed meat products (frankfurter sausage, loin ham, bacon, luncheon meat, and pressed ham (Korean-style luncheon pork)) were purchased from a local market. Frankfurter sausage and bacon were used as such, whereas loin ham, luncheon meat, and pressed ham were cut into 1 cm thickness. Each product was divided into four groups: fresh (control), pan roasting (P), oven grilling (O), and microwave heating (M). The fresh samples were analyzed immediately after cooking, whereas the cooked samples were stored at refrigerated temperature (4 °C) and reheated after 3 and 6 days using the same cooking method or microwave heating, to simulate restaurant preparation. The treatment groups were the following: pan roasting-pan roasting (PP), pan roasting-microwave heating (PM), oven grilling-oven grilling (OO), oven grilling-microwave heating (OM), and microwave heating-microwave heating (MM). Samples for pan roasting (P) were roasted on an electric grill pan (Excel 10 Electric Grill, Tefal Sa, France) at 180 °C until the internal temperature of the samples reached 70 °C. After 3 and 6 days of storage, reheating by pan roasting was done at 180 °C for 5 min (PP) whereas microwave heating was performed in a microwave oven (M-M270TC, LG Electronics, Korea) with 700 W power for 2 min (PM). The samples were cooked by oven grilling in a convection oven (GR-643HT, Tong Yang Magic, Korea) at 150 °C until the internal temperature reached 70 °C. After 3 and 6 days of storage, reheating by oven grilling was done at 150 °C for 10 min (OO) using the same oven, whereas microwave heating was performed in a microwave oven with 700 W power for 2 min (OM). For the MM group, the samples were cooked in a microwave oven for 10 min and reheated in a microwave oven for 2 min, after 3 and 6 days of storage.

### Determination of total cholesterol

The fat (0.5 g) was diluted in 10 mL of freshly prepared methanolic potassium hydroxide solution (1 M) and 1 g of sea sand was added. The mixture was heated for 25 min and the supernatant was transferred with a pipette into a 25 mL volumetric flask. The residue was boiled with 6 mL of isopropanol under reflux condenser for 5 min, and the solution was collected, cooled, and diluted to the mark with isopropanol. The turbid solutions were filtered through a Whatman No. 1 filter paper (Whatman Inc., Clifton, NJ). The clear aliquot was used for the cholesterol assay following the kit instructions (Cat. No 139050, Boehringer Mannheim, Germany). The blank sample was prepared by mixing 0.4 mL of the extracted sample solution and 5 mL of solution 4 (cholesterol reagent mixture). The sample solution was obtained by mixing 2.5 mL of the extracted sample solution and 0.02 mL of solution 3. The prepared blank and sample solutions were sealed with paraffin film and incubated at 37-40 °C for 60 min. The absorbance values of the blank (A1) and of the sample (A2) were determined using an UV spectrophotometer (UV1601, Shimadzu Co., Japan) at 405 nm. Cholesterol contents (mg/100 g) were calculated using the following equation:

$$ \mathrm{Cholesterol}\ \mathrm{content}\ \left(\mathrm{mg}/100\ \mathrm{g}\right)=\left[0.711\times \left(\mathrm{A}2-\mathrm{A}1\right)/\mathrm{sample}\ \mathrm{weight}\left(\mathrm{g}\right)\right]\times 100\times 25 $$

### Cholesterol Oxidation Products (COPs)

Cholesterol oxidation products were analyzed according to the method of Lee et al. [17]. For the separation of COPs, a solid-phase column was prepared [18, 19] by mixing silicic acid, Celite 545, and CaHPO_4_^.^2H_2_O (10:9:1, wt/wt/wt) with 30 mL chloroform, and the mixture was packed in a glass column (12 mm × 30 cm). The prepared column was repeatedly prewashed with 5 mL of hexane before sample application. The total lipids were extracted following the method of Folch et al. [20]. The lipid sample (0.2 g) was dissolved in 2 mL hexane:ethyl acetate (100:2, v/v) and applied to a prewashed column. The sample container was washed twice with 2 mL hexane:ethyl acetate and the wash solvent was applied to the column. Neutral lipid and cholesterol (phospholipids) were removed by adding 50 mL of solvent I (CHCl_3_:CH_3_OH = 2:1, v/v) and 60 mL of solvent II (hexane:ethyl acetate = 4:1, v/v). Solvent III (40 mL, acetone:ethyl acetate:methanol = 10:10:1, v/v/v) was used at a 1 mL/min flow rate to elute the COPs. The collected solutions were dried on a 50 °C hot plate with nitrogen gas flushing. The dried extracts were derivatized by heating at 80 °C for 1 h in the presence of 200 μL pyridine and 100 μL sylon-bis(trimethylsilyl)trifluoroacetamide + 1 % trimethylchlorosilane. The COPs were analyzed using a gas chromatograph (HP 5890 plus) equipped with an on-column capillary injector and a flame ionization detector. Identification of cholesterol and COPs was based on the comparison of the retention times of the samples with those of the standards, on co-chromatography, and on the characteristics of the absorption spectra.

### Statistical analysis

Statistical analysis was performed by one-way analysis of variation (ANOVA) using SAS software (SAS, Release 8.01, SAS Institute Inc., Cary, NC), and Duncan’s multiple range test was employed to differentiate the significance among mean values.

## Results and discussion

### Total fat and cholesterol content

Total fat and cholesterol contents of processed meat products cooked and reheated by different methods after 3 and 6 days of storage at 4 °C are shown in Tables 1 and 2. The highest fat content was found in fresh bacon (48.5 %), followed by sausages (24.7 %), luncheon meats (23.6 %), and pressed ham (16.3 %), and the lowest fat was found in fresh loin meat (8.3 %). The fat content of meat products varied significantly as a result of cooking and reheating as well as during storage. Bacon showed non-significant changes upon cooking and reheating; however, a considerable reduction in fat content was observed after storing the product for 6 days. The cholesterol content of fresh samples did not vary significantly during storage at 4 °C for 6 days (Table 2). Cooking and reheating of meat products greatly affect their cholesterol content, and maximum reduction in cholesterol was observed for oven grilling and microwave cooking and reheating. Among the tested samples, the highest cholesterol content (201.70 mg/100 g) was observed in bacon, and the lowest (76.00 mg/100 g) in pressed ham. In the present study, the wide range of cholesterol content from pressed ham to bacon is probably due to a high variability in the original samples. Rodriguez-Estrada et al. [2] reported the presence of higher amounts of cholesterol in fresh samples compared with the cooked samples, and attributed these differences to the loss of cholesterol during the cooking process. Cooking and reheating resulted in the oxidation of cholesterol and production of COPs. These findings are in agreement with the results obtained by Freitas et al. [16], who reported a decrease in the cholesterol content of fish fillets and an increase in COP formation upon cooking. Badiani et al. [21] reported that cooking induced a remarkable decrease in moisture content, which varies depending on the different cooking methods, leading to various cholesterol levels due to the differences in cooking and reheating techniques. The results obtained in the current study also confirm the findings of Baggio and Bragagnolo [22], who observed that storage time did not alter the cholesterol content of processed meat samples.

**Table 1:** Changes of the crude fat content (%) of processed meat products in fresh, cooked, and re-heated at day 3 and 6 of storage

Sample	Cooking method	Re-heating (day)		
		0	3	6
Sausage	Fresh	24.7 ± 0.09^AB^	24.5 ± 0.66^AB^	24.1 ± 0.53^A^
	PP	24.6 ± 0.0^Ba^	23.6 ± 0.01^BCb^	23.5 ± 0.10^Bb^
	PM	24.6 ± 0.0^Ba^	23.0 ± 0.98^Cb^	22.8 ± 0.13^Cb^
	OO	25.2 ± 0.78^Aa^	24.7 ± 0.50^Aa^	23.8 ± 0.01^ABb^
	OM	25.2 ± 0.78^Aa^	24.0 ± 0.11^ABCb^	23.7 ± 0.10^ABb^
	MM	23.3 ± 0.06^Ca^	23.0 ± 0.11^Cb^	23.0 ± 0.12^Cb^
Loin ham	Fresh	8.3 ± 0.09^A^	8.1 ± 0.52^A^	8.1 ± 0.56^A^
	PP	5.7 ± 0.01^Ba^	5.5 ± 0.01^Bb^	5.3 ± 0.13^Bc^
	PM	5.7 ± 0.01^Ba^	5.4 ± 0.01^Bb^	4.8 ± 0.19^Bc^
	OO	6.8 ± 0.01^Ba^	5.8 ± 0.03^Bb^	4.9 ± 0.04^Bc^
	OM	6.8 ± 0.01^Ba^	5.4 ± 0.10^Bb^	5.0 ± 012^Bc^
	MM	5.5 ± 0.01^Bb^	5.8 ± 0.11^Ba^	5.1 ± 0.08^Bc^
Bacon	Fresh	48.5 ± 0.51	48.7 ± 0.84	48.6 ± 1.00
	PP	49.0 ± 0.57^a^	47.7 ± 0.01^b^	46.6 ± 0.09^c^
	PM	49.0 ± 0.57^a^	47.5 ± 0.71^b^	44.6 ± 0.56^c^
	OO	47.0 ± 0.0^a^	45.9 ± 0.29^b^	45.7 ± 0.09^b^
	OM	47.0 ± 0.01^b^	48.6 ± 0.38^a^	43.8 ± 0.10^c^
	MM	48.8 ± 0.62^a^	45.8 ± 0.02^b^	42.9 ± 0.10^c^
Luncheon meat	Fresh	23.6 ± 1.10^AB^	23.2 ± 0.71^A^	23.2 ± 0.69^BC^
	PP	24.0 ± 0.07^Ab^	27.1 ± 0.11^Aa^	27.2 ± 0.14^Aa^
	PM	24.0 ± 0.07^Ab^	23.6 ± 0.08^Ac^	25.9 ± 0.08^Aba^
	OO	24.0 ± 0.11^Ac^	26.8 ± 0.10^Aa^	24.6 ± 0.21^ABb^
	OM	24.0 ± 0.11^Ba^	20.3 ± 0.10^Bb^	19.2 ± 0.10^Cc^
	MM	23.4 ± 0.09^AB^	24.3 ± 0.08^A^	23.5 ± 0.12^ABC^
Press Ham	Fresh	16.3 ± 0.10^B^	16.4 ± 0.31^B^	16.2 ± 0.26^B^
	PP	18.2 ± 0.09^Ac^	21.4 ± 0.01^Aa^	19.6 ± 0.08^Ab^
	PM	18.2 ± 0.09^Ac^	18.8 ± 0.10^Ab^	19.8 ± 0.13^Aa^
	OO	20.1 ± 0.08^Aa^	18.4 ± 0.16^Ab^	19.9 ± 0.55^Aa^
	OM	20.1 ± 0.08^Ab^	16.6 ± 0.07^Ac^	21.2 ± 0.01^Aa^
	MM	20.3 ± 0.07^Ac^	21.4 ± 0.12^Aa^	20.6 ± 0.08^Ab^

Means ± SE with different superscript in the same row (^a-c^) and column (^A-C^) differ significantly (*p* < 0.05)

Pan roasting (cooking) + pan roasting (re-heating), PP; pan roasting (cooking) + microwaving (re-heating), PM; oven grilling (cooking) + oven grilling (re-heating), OO; oven grilling (cooking) + microwaving (re-heating), OM; microwaving (cooking) + microwaving (re-heating), MM

**Table 2:** Changes of cholesterol content (mg/100 g) of meat products in fresh, cooked, and re-heated at day 3 and 6 of storage

Sample	Cooking method	Re-heating (day)		
		0	3	6
Sausage	Fresh	96.2 ± 11.52	95.3 ± 11.24	94.7 ± 10.99^C^
	PP	103.3 ± 6.57^b^	86.0 ± 4.63^c^	137.9 ± 4.78^Aa^
	PM	103.3 ± 6.57^b^	84.6 ± 5.11^c^	119.5 ± 3.64^Ba^
	OO	93.0 ± 4.22	88.3 ± 11.88	91.5 ± 1.14^C^
	OM	93.0 ± 4.22^a^	74.3 ± 6.25^b^	93.0 ± 3.02^Ca^
	MM	93.4 ± 7.07	90.1 ± 4.07	91.2 ± 4.05^C^
Loin ham	Fresh	91.7 ± 7.93^B^	91.9 ± 7.41^A^	91.9 ± 7.65^A^
	PP	54.6 ± 1.61^Cb^	47.8 ± 3.20^Cc^	89.8 ± 2.82^Aa^
	PM	54.6 ± 1.61^Cc^	71.8 ± 2.54^Ba^	64.3 ± 3.79^Cb^
	OO	101.4 ± 6.03^Aa^	52.5 ± 1.54^Cc^	85.6 ± 2.86^ABb^
	OM	101.4 ± 6.03^Aa^	55.6 ± 3.04^Cc^	79.8 ± 0.91^Bb^
	MM	45.1 ± 1.83^Da^	49.4 ± 4.62^Ca^	29.5 ± 0.37^Db^
Bacon	Fresh	201.70 ± 7.95^A^	201.2 ± 8.68^BC^	200.8 ± 7.56^B^
	PP	155.4 ± 8.95^Bc^	205.7 ± 7.61^ABb^	238.0 ± 9.32^Aa^
	PM	155.4 ± 8.95^Bb^	232.4 ± 18.58^Aa^	218.1 ± 18.81^Aba^
	OO	140.8 ± 23.0^Bb^	158.8 ± 27.23^Dab^	198.3 ± 9.19^Ba^
	OM	140.8 ± 23.0^B^	173.9 ± 18.08^CD^	168.1 ± 10.21^C^
	MM	151.6 ± 4.84^B^	174.0 ± 6.48^CD^	163.5 ± 12.63^C^
Luncheon meat	Fresh	106.2 ± 5.06^A^	106.1 ± 4.16^A^	106.2 ± 4.83^BC^
	PP	92.8 ± 9.80^B^	94.2 ± 14.45^AB^	81.8 ± 5.29^C^
	PM	92.8 ± 9.80^B^	98.3 ± 18.34^AB^	125.3 ± 15.61^B^
	OO	91.8 ± 2.15^Ba^	77.1 ± 3.94^Bb^	80.1 ± 2.27^Ca^
	OM	91.8 ± 2.15^B^	91.7 ± 6.07^AB^	121.8 ± 31.64^B^
	MM	79.7 ± 6.93^Bc^	111.1 ± 13.55^Ab^	214.4 ± 6.50^Aa^
Press ham	Fresh	76.0 ± 7.30	75.5 ± 6.85^B^	75.4 ± 6.61^B^
	PP	81.0 ± 4.38^a^	70.6 ± 3.84^Bb^	53.2 ± 4.35^BCc^
	PM	81.0 ± 4.38	92.3 ± 11.04^A^	52.7 ± 43.23^BC^
	OO	67.9 ± 9.10	65.4 ± 9.43^B^	69.6 ± 3.99^B^
	OM	67.9 ± 9.10^a^	59.4 ± 11.87^Ba^	31.1 ± 1.71^Cb^
	MM	67.8 ± 12.47^b^	62.2 ± 6.01^Bb^	191.5 ± 9.40^Aa^

Means ± SE with different superscript in the same row (^a-c^) and column (^A-C^) differ significantly (*p* < 0.05)

Pan roasting (cooking) + pan roasting (re-heating), PP; pan roasting (cooking) + microwaving (re-heating), PM; oven grilling (cooking) + oven grilling (re-heating), OO; oven grilling (cooking) + microwaving (re-heating), OM; microwaving (cooking) + microwaving (re-heating), MM

### Cooking methods and COP formation

The data expressed in Table 3 shows the impact of the cooking methods on the formation of COPs in fresh meat products. 7β-OH was observed in sausages and bacon for all cooking methods, whereas only microwave cooking produces 7β-OH in loin ham. However, 7β-OH was not detected in luncheon meat and pressed ham after cooking by all methods. The highest level of 7β-OH (360.54 μg/100 g) was observed in sausages cooked by oven grilling. A low level of 25-OH was found in fresh as well as in cooked loin ham and in oven grilled luncheon meat. A high 25-OH level was observed in sausages cooked by microwave. Cholestanetriol was detected in microwaved sausage (171.6 μg/100 g), pan-roasted bacon (184.2 μg/100 g), and fresh (153.8 μg/100 g) and oven grilled luncheon meat (256.9 μg/100 g). Low levels of α-epoxide were found in fresh, pan roasted, and microwaved sausages, and in oven grilled loin ham and luncheon meat. 20α-OH and 7-keto were not detected in any of the meat products for all the tested cooking methods.

**Table 3:** The amount of cholesterol oxidation products (μg/100 g) of meat products with different cooking methods

Sample	Cooking method	Cholesterol Oxidation Products						COPs/cholesterol (%)
		7β-OH	20α-OH	25-OH	Triol	α-epoxide	7-keto	
Sausage	Fresh	n.d.	n.d.	n.d.	n.d.	20.2 ± 34.94	n.d.	0.02 ± 0.04
	P	58.3 ± 100.98	n.d.	n.d.	n.d.	29.9 ± 51.69	n.d.	0.08 ± 0.14
	O	360.5 ± 624.48	n.d.	n.d.	n.d.	-.	n.d.	0.36 ± 0.63
	M	204.3 ± 228.85	n.d.	359.8 ± 598.90	171.6 ± 297.14	17.4 ± 30.08	n.d.	0.73 ± 0.71
Loin ham	Fresh	n.d.	n.d.	43.0 ± 74.39	n.d.	n.d.	n.d.	0.05 ± 0.08
	P	n.d.	n.d.	5.5 ± 9.49	n.d.	n.d.	n.d.	0.01 ± 0.01
	O	n.d.	n.d.	22.0 ± 38.01	n.d.	11.6 ± 20.12	n.d.	0.04 ± 0.07
	M	24.6 ± 33.33	n.d.	127.3 ± 171.25	n.d.	n.d.	n.d.	0.16 ± 0.21
Bacon	Fresh	n.d.	n.d.	n.d.	n.d.	n.d.	n.d.	0.00 ± 0.00
	P	134.2 ± 26.97	n.d.	n.d.	184.2 ± 319.09	n.d.	n.d.	0.16 ± 0.15
	O	110.6 ± 191.50	n.d.	n.d.	n.d.	n.d.	n.d.	0.05 ± 0.09
	M	304.4 ± 527.25	n.d.	n.d.	n.d.	n.d.	n.d.	0.16 ± 0.27
Luncheon meat	Fresh	n.d.	n.d.	n.d.	153.8 ± 266.35	n.d.	n.d.	0.15 ± 0.26
	P	n.d.	n.d.	n.d.	n.d.	n.d.	n.d.	0.00 ± 0.00
	O	n.d.	n.d.	30.9 ± 53.49	256.9 ± 445.03	50.7 ± 87.73	n.d.	0.32 ± 0.48
	M	n.d.	n.d.	n.d.	n.d.	n.d.	n.d.	0.00 ± 0.00
Press ham	Fresh	n.d.	n.d.	n.d.	n.d.	n.d.	n.d.	0.00 ± 0.00
	P	n.d.	n.d.	n.d.	n.d.	n.d.	n.d.	0.00 ± 0.00
	O	n.d.	n.d.	n.d.	n.d.	n.d.	n.d.	0.00 ± 0.00
	M	n.d.	n.d.	n.d.	n.d.	n.d.	n.d.	0.00 ± 0.00

Pan roasting, P; oven grilling, O; microwaving, M; 7β-hydroxycholesterol, 7β-OH; 20α-hydroxycholesterol, 20α-OH; 25-hydroxycholesterol, 25-OH; cholestane-3β,5α,6β-triol, triol; cholesterol-5α,6α-epoxide, α-epoxide; 7-ketocholesterol, 7-keto

The ratio between the total amount of COPs and cholesterol in the different meat products cooked by different methods was found to be very low (0–0.73 %). The highest value was observed in sausages cooked by microwave (0.73 %), whereas bacon contained the highest cholesterol content (Table 2) and a low total COPs (0.16 %) (Table 3). The presence of a higher amount of 7β-OH in cooked meat products is in agreement with the previous finding of Du et al. [23], who observed a higher level of 7α-OH and 7β-OH compared with other COPs in cooked turkey meat. Broncano et al. [24] investigated different cooking methods and their role in lipid oxidation and in the formation of COPs, and reported the presence of higher levels of 7α-OH and 7β-OH than other COPs. The absence or lower level of triol, α-epoxide, 20α-OH, and 7-keto than 7β-OH is in accordance with the findings of Grau et al. [25] and Ghiretti et al. [26]. The higher COP formation owing to microwaving confirms the results of Dominguez et al. [14], who, upon cooking foal meat using roasting, grilling, microwaving, and frying, detected the highest lipid oxidation in microwaved samples. Serra et al. [27] reported 7β-OH and 7-keto as the primary products of cholesterol oxidation, and α- and β-epoxides as the secondary oxidation products.

### Storage, reheating, and COP formation

The cooked meat products stored at 4 °C and reheated after 3 and 6 storage days were assessed for the formation of COPs (Tables 4 and 5). It is evident from the data presented in Table 4 that the COPs observed in cooked products increased significantly upon reheating after 3 storage days. However, 20α-OH was not found in any of the samples reheated by different methods, and 7-keto was detected only in pan roasted luncheon meat reheated by microwave. 7β-OH, 25-OH, and α-epoxide were present in greater amounts than other COPs and were found namely in sausages, loin ham, and bacon. 7β-OH was detected in all samples of sausages, loin ham, and bacon reheated after 3 days by different methods. Similarly, 25-OH and α-epoxide were present in all reheated samples of loin ham. Pressed ham samples also showed cholesterol oxides after 3 days of storage, whereas no oxides were detected after cooking. 7β-OH was found to be present in the highest quantity (497 μg/100 g) pressed ham cooked by oven grilling and reheated with the same method after 3 days of storage. The ratio total amount of COPs/cholesterol in meat products increased relatively after 3 days of storage (0–0.86 %). The data presented in Table 4 shows that the highest ratio (0.86 %) was observed in cooked sausage reheated by microwave.

**Table 4:** The amount of cholesterol oxidation products (μg/100 g) of meat products cooked with different methods and re-heated by microwaving at day 3 of storage at 4 °C

Sample	Cooking method	Cholesterol Oxidation Products						COPs/cholesterol (%)
		7β-OH	20α-OH	25-OH	Triol	α-epoxide	7-keto	
Sausage	Fresh	12.7 ± 14.14	n.d.	n.d.	n.d.	n.d.	n.d.	0.01 ± 0.01
	PP	155.1 ± 268.60	n.d.	n.d.	283.0 ± 49.01	n.d.	n.d.	0.42 ± 0.42
	PM	42.6 ± 73.81	n.d.	396.1 ± 686.09	n.d.	15.7 ± 27.24^B^	n.d.	0.46 ± 0.69
	OO	300.3 ± 520.14	n.d.	n.d.	n.d.	n.d.	n.d.	0.34 ± 0.59
	OM	147.3 ± 140.60	n.d.	278.2 ± 481.78	87.8 ± 152.00	n.d.	n.d.	0.57 ± 0.49
	MM	55.2 ± 95.63	n.d.	381.2 ± 660.22	n.d.	315.0 ± 320.37^B^	n.d.	0.86 ± 1.07
Loin ham	Fresh	n.d.	n.d.	43.5 ± 75.38	n.d.	26.0 ± 45.07^B^	n.d.	0.07 ± 0.13
	PP	22.9 ± 29.78	n.d.	196.5 ± 153.41	n.d.	125.2 ± 216.91^B^	n.d.	0.64 ± 0.63
	PM	9.9 ± 17.08	n.d.	13.5 ± 23.38	26.4 ± 45.70	19.4 ± 23.69^B^	n.d.	0.13 ± 0.10
	OO	8.9 ± 8.05	n.d.	58.7 ± 101.69	54.6 ± 94.51	77.1 ± 124.63^B^	n.d.	0.20 ± 0.20
	OM	8.6 ± 14.85	n.d.	25.0 ± 43.34	16.4 ± 28.44	79.7 ± 138.02^B^	n.d.	0.13 ± 0.16
	MM	56.6 ± 98.02	n.d.	241.6 ± 213.09	n.d.	34.2 ± 59.23^B^	n.d.	0.73 ± 0.64
Bacon	Fresh	n.d.	n.d.	n.d.	n.d.	n.d.	n.d.	0.00 ± 0.00
	PP	899.1 ± 1557.30	n.d.	n.d.	245.6 ± 425.41	n.d.	n.d.	0.71 ± 0.84
	PM	120.7 ± 209.00	n.d.	n.d.	109.7 ± 189.93	n.d.	n.d.	0.14 ± 0.13
	OO	94.7 ± 164.09	n.d.	247.1 ± 427.91	n.d.	751.2 ± 909.52^A^	n.d.	0.81 ± 0.95
	OM	111.6 ± 193.21	n.d.	n.d.	212.5 ± 368.04	n.d.	n.d.	0.25 ± 0.24
	MM	589.4 ± 1020.48	n.d.	667.2 ± 1155.64	n.d.	n.d.	n.d.	0.84 ± 0.73
Luncheon meat	Fresh	n.d.	n.d.	n.d.	156.6 ± 271.45	n.d.	n.d.	0.15 ± 0.27
	PP	n.d.	n.d.	n.d.	n.d.	n.d.	n.d.	0.00 ± 0.00
	PM	150.5 ± 260.58	n.d.	52.6 ± 91.14	205.8 ± 356.42	37.5 ± 37.68^B^	26.3 ± 45.51	0.48 ± 0.44
	OO	n.d.	n.d.	10.7 ± 18.51	107.1 ± 185.42	n.d.	n.d.	0.13 ± 0.22
	OM	87.7 ± 151.86	n.d.	95.1 ± 164.77	n.d.	n.d.	n.d.	0.20 ± 0.17
	MM	231.2 ± 440.37	n.d.	n.d.	n.d.	n.d.	n.d.	0.32 ± 0.55
Press ham	Fresh	n.d.	n.d.	48.1 ± 76.50	n.d.	n.d.	n.d.	0.07 ± 0.11
	PP	n.d.	n.d.	n.d.	168.4 ± 162.53	n.d.	n.d.	0.22 ± 0.21
	PM	n.d.	n.d.	112.2 ± 194.33	n.d.	n.d.	n.d.	0.15 ± 0.26
	OO	497.0 ± 860.89	n.d.	n.d.	n.d.	n.d.	n.d.	0.63 ± 1.10
	OM	n.d.	n.d.	91.8 ± 159.05	n.d.	n.d.	n.d.	0.15 ± 0.26
	MM	n.d.	n.d.	106.5 ± 184.50	n.d.	n.d.	n.d.	0.13 ± 0.22

Means ± SE with different superscript in the same column (^A-C^) differ significantly

Pan roasting (cooking) + pan roasting (re-heating), PP; pan roasting (cooking) + microwaving (re-heating), PM; oven grilling (cooking) + oven grilling (re-heating), OO; oven grilling (cooking) + microwaving (re-heating), OM; microwaving (cooking) + microwaving (re-heating), MM; 7β-hydroxycholesterol, 7β-OH; 20α-hydroxycholesterol, 20α-OH; 25-hydroxycholesterol, 25-OH; cholestane-3β,5α,6β-triol, triol; cholesterol-5α,6α-epoxide, α-epoxide; 7-ketocholesterol, 7-keto

**Table 5:** The amount of cholesterol oxidation products (μg/100 g) of meat products cooked with various methods and re-heated by microwaving at day 6 of storage at 4 °C

Sample	Cooking method	Cholesterol Oxidation Products						COPs/cholesterol (%)
		7β-OH	20α-OH	25-OH	Triol	α-epoxide	7-keto	
Sausage	Fresh	35.3 ± 0.49	n.d.	13.3 ± 14.56	41.8 ± 68.60	35.1 ± 47.79	n.d.	0.13 ± 0.02^C^
	PP	127.3 ± 141.26	n.d.	56.6 ± 97.99	43.8 ± 75.89	20.2 ± 34.94	n.d.	0.24 ± 0.12^C^
	PM	51.1 ± 88.42	n.d.	303.9 ± 526.40	72.9 ± 126.26	n.d.	n.d.	0.39 ± 0.50^C^
	OO	68.0 ± 117.73	n.d.	36.9 ± 63.91	125.9 ± 218.12	n.d.	n.d.	0.25 ± 0.15^C^
	OM	194.3 ± 242.03	n.d.	29.9 ± 51.78	913.8 ± 1582.68	130.0 ± 225.16	n.d.	1.32 ± 1.81^BC^
	MM	339.5 ± 437.60	n.d.	52.0 ± 89.99	120.6 ± 187.71	n.d.	n.d.	0.54 ± 0.26^C^
Loin ham	Fresh	n.d.	n.d.	43.7 ± 75.79	n.d.	25.8 ± 44.74	n.d.	0.07 ± 0.13^C^
	PP	14.1 ± 24.33	n.d.	147.2 ± 232.12	36.0 ± 62.31	119.7 ± 167.93	n.d.	0.57 ± 0.70^C^
	PM	9.3 ± 16.11	n.d.	n.d.	n.d.	36.7 ± 63.53	n.d.	0.09 ± 0.11^C^
	OO	15.6 ± 26.95	n.d.	114.9 ± 156.50	79.7 ± 138.07	191.0 ± 204.85	n.d.	0.39 ± 0.30^C^
	OM	17.2 ± 29.86	n.d.	243.7 ± 388.99	125.2 ± 216.89	188.8 ± 178.94	37.7 ± 65.30	0.59 ± 0.48^C^
	MM	23.5 ± 40.75	n.d.	131.33 ± 187.97	-	50.8 ± 87.99	n.d.	0.45 ± 0.44^C^
Bacon	Fresh	n.d.	n.d.	n.d.	n.d.	n.d.	n.d.	0.00 ± 0.00^C^
	PP	141.4 ± 244.96	n.d.	n.d.	164.2 ± 284.39	n.d.	n.d.	0.19 ± 0.17^C^
	PM	98.1 ± 069.85	n.d.	n.d.	106.9 ± 105.64	238.0 ± 412.15	n.d.	0.28 ± 0.30^C^
	OO	98.7 ± 100.05	n.d.	n.d.	228.2 ± 395.25	n.d.	n.d.	0.25 ± 0.24^C^
	OM	75.9 ± 131.39	n.d.	1718.6 ± 2976.73	2646.3 ± 4583.49	358.7 ± 470.27	n.d.	3.30 ± 2.29^A^
	MM	91.1 ± 157.75	52.8 ± 91.46	n.d.	n.d.	347.6 ± 602.05	n.d.	0.32 ± 0.31^C^
Luncheon meat	Fresh	n.d.	n.d.	n.d.	154.4 ± 267.47	69.2 ± 77.20	n.d.	0.22 ± 0.26^C^
	PP	n.d.	n.d.	n.d.	n.d.	n.d.	n.d.	0.00 ± 0.00^C^
	PM	122.8 ± 212.66	47.9 ± 82.89	n.d.	n.d.	n.d.	n.d.	0.21 ± 0.36^C^
	OO	n.d.	n.d.	82.1 ± 142.08	158.8 ± 274.96	n.d.	n.d.	0.26 ± 0.44^C^
	OM	888.6 ± 1539.04	22.4 ± 38.75	84.4 ± 146.13	n.d.	102.0 ± 176.73	n.d.	1.20 ± 1.56^BC^
	MM	142.2 ± 246.24	27.2 ± 47.47	n.d.	n.d.	n.d.	n.d.	0.23 ± 0.40
Press ham	Fresh	n.d.	n.d.	48.1 ± 76.50	n.d.	73.9 ± 111.53	n.d.	0.16 ± 0.11
	PP	n.d.	19.7 ± 34.07	96.0 ± 138.77	n.d.	26.4 ± 45.66	18.3 ± 31.77	0.20 ± 0.18
	PM	n.d.	n.d.	n.d.	449.8 ± 779.10	164.8 ± 285.46	n.d.	0.81 ± 1.40^C^
	OO	1017.2 ± 1761.78	n.d.	n.d.	n.d.	n.d.	n.d.	1.63 ± 2.83^ABC^
	OM	1175.2 ± 2012.57	n.d.	n.d.	461.2 ± 798.81	164.6 ± 285.09	n.d.	2.87 ± 2.78^AB^
	MM	n.d.	37.7 ± 65.31	n.d.	n.d.	n.d.	177.7 ± 307.76	0.36 ± 0.46^C^

Means ± SE with different superscript in the same column (^A-C^) differ significantly

Pan roasting (cooking) + pan roasting (re-heating), PP; pan roasting (cooking) + microwaving (re-heating), PM; oven grilling (cooking) + oven grilling (re-heating), OO; oven grilling (cooking) + microwaving (re-heating), OM; microwaving (cooking) + microwaving (re-heating), MM; 7β-hydroxycholesterol, 7β-OH; 20α-hydroxycholesterol, 20α-OH; 25-hydroxycholesterol, 25-OH; cholestane-3β,5α,6β-triol, triol; cholesterol-5α,6α-epoxide, α-epoxide; 7-ketocholesterol, 7-keto

Generally, the amount of COPs in samples reheated after 6 days of storage at 4 °C (Table 5) was higher than that in samples reheated after 3 days of storage, suggesting that increasing the storage time after cooking increased the COP content in processed meat products. 20α-OH was not detected in any of the cooked samples nor in those reheated after 3 days; however, it appeared in samples reheated after 6 days of storage. A similar trend was also observed for 7-keto. The ratio between the total amount of COPs and cholesterol in meat products also increased after 6 days of storage (0.00-3.30 %), with the highest ratio in bacon cooked by oven grilling and reheated by microwave. The concentration of COPs in pressed ham was not detected after cooking and increased significantly upon reheating; the highest total amount of COPs/cholesterol ratios were observed in OM (2.87 %) and OO (1.63 %). Our findings are in line with previous investigations, indicating that storage of cooked meat results in an increase in COP concentration upon reheating. Monahan et al. [28] reported that the rate of cholesterol oxidation in pork products significantly increased during storage after cooking. A higher level of lipid oxidation was found by El-Alim et al. [29] in microwave cooked meat patties after 7 storage days at 4 °C. Hu et al. [15] also observed significantly higher COP levels in microwave cooked samples compared with the COP content in samples cooked by other methods. Processed meat initially free of COPs showed a minor increase in COP content during storage at −4 °C for 4 weeks and a reduction of 15 to 24 % in free cholesterol [30]. Juarez et al. [31] investigated the impact of different cooking methods on buffalo meat composition. Greater oxidative changes were observed in boiled and grilled samples, based on higher TBARS (thiobarbituric acid reactive substances) values. Ferioli et al. [32] evaluated lipid and cholesterol oxidation in fresh and cooked minced beef stored at 4 °C; the COP content increased by 6 times after 2 weeks of storage. They also revealed that the increase in the COP content was higher in cooked meat than in fresh samples. Our investigation confirms these results, as a significant increase in the COP content was detected after refrigerated storage of the cooked meat samples.

## Conclusions

Cooking of processed meat products resulted in a decrease of lipids and total cholesterol and in an increase in COP formation. Microwaving and oven grilling led to the production of higher amounts of COPs as compared with other cooking methods. The refrigerated storage of cooked products and subsequent reheating remarkably increased the amount of COPs in processed meat products. However, this increase was found to be lower in fresh products than in cooked products.

## Abbreviations

COPs: Cholesterol oxidation products
BHT: Butylated hydroxyl toluene
GC: Gas chromatography
SAS: Statistical analysis system
7β-OH: 7β-hydroxycholesterol
20α-OH: 7α-hydroxy-cholesterol
25-OH: 25-hydroxycholesterol
Triol: Cholestanetriol
α-epoxide: 5,6α-epoxycholesterol
7-keto: 7-ketocholesterol
